# Platelet Anti-Aggregant Activity and Bioactive Compounds of Ultrasound-Assisted Extracts from Whole and Seedless Tomato Pomace

**DOI:** 10.3390/foods9111564

**Published:** 2020-10-28

**Authors:** Anibal Concha-Meyer, Iván Palomo, Andrea Plaza, Adriana Gadioli Tarone, Mário Roberto Maróstica Junior, Sonia G. Sáyago-Ayerdi, Eduardo Fuentes

**Affiliations:** 1 Facultad de Ciencias Agrarias, Universidad de Talca, Talca 3460000, Chile; anibal.concha@utalca.cl; 2 Centro de Estudios en Alimentos Procesados (CEAP), CONICYT-Regional, Gore Maule, R09I2001, Talca 3460000, Chile; aplaza@ceap.cl; 3 Thrombosis Research Center, Medical Technology School, Department of Clinical Biochemistry and Immunohaematology, Faculty of Health Sciences, Universidad de Talca, Talca 3460000, Chile; 4 LANUM (Laboratory of Nutrition and Metabolism), FEA (School of Food Engineering), UNICAMP (University of Campinas), Rua Monteiro Lobato, 80, Campinas 13083-862, Brazil; dricagt@gmail.com (A.G.T.); mmarosti@unicamp.br (M.R.M.J.); 5 Tecnologico Nacional de Mexico, Instituto Tecnologico de Tepic, Av Tecnológico 2595, Col Lagos del Country, Tepic 63175, Nayarit Mexico, Mexico; ssayago@ittepic.edu.mx

**Keywords:** tomato pomace, extraction, platelet, ultrasound, functional ingredient

## Abstract

Tomato paste production generates a residue known as tomato pomace, which corresponds to peels and seeds separated during tomato processing. Currently, there is an opportunity to use tomato pomace to obtain a functional extract with antithrombotic properties, such as platelet anti-aggregant activity. The aim of this study was to evaluate the yield and inhibitory activity of different extracts of tomato pomace on in vitro platelet aggregation, comparing this activity with commercial cardioprotective products, and quantify bioactive compounds. Aqueous or ethanolic/water (1:1) extracts of whole tomato pomace, seedless tomato pomace, tomato pomace supplemented with seeds (50% and 20%), and only seeds were obtained with different ultrasound-assisted extraction times. The inhibition of platelet aggregation was evaluated using a lumi-aggregometer. The quantification of bioactive compounds was determined by HPLC-MS. From 5 g of each type of tomato pomace sample, 0.023–0.22 g of a dry extract was obtained for the platelet aggregation assay. The time of sonication and extraction solvent had a significant role in platelet anti-aggregant activity of some extracts respect the control. Thus, the most active extracts decreased adenosine diphosphate (ADP)-induced platelet aggregation from 87 ± 6% (control) to values between 26 ± 6% and 34 ± 2% (*p* < 0.05). Furthermore, different ultrasound-assisted extraction conditions of tomato pomace fractions had varied concentration of flavonoids and nucleosides, and had an effect on extract yield.

## 1. Introduction

Cardiovascular disease (CVD) is the number one cause of death worldwide since more people die annually (≈17 million) from CVD than from any other cause [1]. In Chile, CVD causes 27% of deaths [2]. In this context, the adherence to a diet rich in fruit and vegetables was associated with a decrease of all-cause mortality among individuals with CVD [3,4,5]. Furthermore, tomato (*Lycopersicon esculentum*) has been one of the most powerful vegetables associated with the reduction of this type of disease [6,7,8,9,10]. Emerging epidemiological and interventional data support the connection between higher tomato consumption and a lower risk of CVD. Additionally, tomato has the lowest uric acid content of any fruit and vegetable [11,12]. The latter is important because a high uric acid level in the body can cause various health issues like arthritis [13].

Tomato is an important vegetable grown in many countries across the world for fresh markets and multiple processed forms. The worldwide production of processed tomato in 2017 was 38 million tons [14]. During the season 2017–2018, Chile produced 918,000 metric tons of industrial tomato and the Maule region represented about 66% of this total [15]. This crop is industrially processed to produce concentrated tomato paste, which is used to formulate products such as ketchup, sauce, puree, and juice. This processing generates a byproduct named tomato pomace, corresponding mostly to peels and seeds that account for 3–5% (wet basis (w.b.)) of fresh tomato [16]. Currently, the tomato pomace has different uses, such as a protein supplement for growing lambs [17], ruminant feeding [18], chemical and nutritional supplementation of crackers [19], and sustainable fabrication of packaging films [20]. Furthermore, if the latter is not possible, this residue is disposed of on agricultural lands causing environmental contamination [21,22]. The results of the proximate composition of tomato pomace, on a dry weight basis, showed a relevant nutritional content of total protein (19.40%, N × 6.25), total fat (12.33%), available carbohydrates (17.15%), total dietary fiber (47.80%), ash (3.32%), sodium (13.20 mg/100 g), and sugars (9.54%) [23]. Meanwhile, tomato seed oil contains high levels of linoleic (54%) and oleic (22%) acids [24]. On the other hand, the main polysaccharides identified in tomato pomace correspond to glucose (40.5 ± 1.2%) and fructose (22.6 ± 2.1%) from total carbohydrates of 382.9 ± 10.3 mg/g dry weight [25]. Tomato pomace is also known as a source of bioactive compounds, such as carotenoids, phenolic compounds, and dietary fiber [26]. Considering the above, there is an opportunity to use tomato pomace to obtain a functional product with antithrombotic properties, such as platelet anti-aggregant activity, that could be useful as an ingredient in healthy foods for CVD prevention [5].

Different extraction procedures have been evaluated to increase the efficiency of tomato pomace compound extraction for pectin, dietary fiber, and carotenoids such as lycopene, including varied solvent extraction by mixing and heating, microwave-assisted extraction, enzymatic extraction, ultrasound-assisted extraction, and ultrasound-assisted treatment combined with subcritical water [27,28,29,30]. Ultrasound is a non-thermal technology that has shown to be particularly effective for improving the extraction of heat-labile compounds [31]. Ultrasound equipment is also commonly available in most analytic laboratories and is used to improve the efficiency of any extraction solvent, thus reducing the extraction times [32]. Furthermore, there is a need to improve the extraction of bioactive compounds, such as flavonoids and nucleosides (adenosine and guanosine), that are present in tomato (*Solanum lycopersicum)* and tomato pomace, which have significant platelet anti-aggregant activity [5,6,33,34]. This work aimed to evaluate the chemical profile and platelet anti-aggregant activity of ultrasound-assisted extracts of different tomato pomace fractions obtained using water or ethanol/water (1:1) as the solvent.

## 2. Materials and Methods

### 2.1. Chemicals

The solvents used were ethanol (Sigma-Aldrich, St. Louis, MO, USA) and type I ultrapure water (18.2 MΩ-cm), which was supplied by the Purelab Classic Elga water system (Labwater/VWS Ltd., London, UK). Adenosine diphosphate (ADP) was used as an agonist in the platelet aggregation assay (Chrono-log, Havertown, PA, USA).

### 2.2. Plant Material

Tomato pomace (mainly peels and seeds), which is a byproduct of the industrial production of tomato paste, was obtained from the company Sugal Chile (Talca, Chile) during the 2016 season. Given the production line of the plant, it was not possible to define specific fruit hybrids that corresponded to the tomato pomace; and it was only possible to identify middle (Sun6366, AB3, and HMX7883) and late (H9665, H7709, and H9997) hybrid tomatoes [35]. Tomato pomace was placed in trays and dried for 48 h at 60 °C in a convection oven (VHC-1A, Ventus Corp., Santiago, Chile); after this, a portion of tomato pomace (22% of seeds) was manually separated into seeds and peels for the preparation of the different extracts; and finally, all parts from tomato pomace were milled and sieved through a 425 µm mesh (No. 40) (TRF 300, TRAPP, Jaraguá do Sul, Brazil). 

### 2.3. Preparation of Extracts

Aqueous or ethanolic/water (1:1) extracts of whole tomato pomace, seedless tomato pomace, tomato pomace supplemented with seeds (50% and 20%), and only seeds were obtained. Briefly, 5 g of each fraction was mixed with 50 mL of water for aqueous extract, and with 25 mL of water and 25 mL of ethanol for ethanolic extracts. An ultrasonic bath of 2.8 L, 220 V, amplitude 100%, and power 90 W (VWR 97043-962, Leicestershire, UK), was used with different sonication time cycles were applied as the extraction process: cycle 1 with one cycle of 20 min, cycle 3 with three cycles of 20 min each with a 10-min pause in between, and cycle 6 with six cycles of 20 min each with a 10-min pause in between. All cycles were controlled each 1 min at a frequency of 35 kHz and a temperature of 45 °C. Then, the extracts were centrifuged at 725× *g* (International Equipment Company IEC, Centra MP4R, Boston, MA, USA) for 5 min to obtain the supernatant, which was frozen at −86 °C for 48 h, then freeze-dried (Operon, FDU 7024, Gimpo, Korea) for 20 h with a cold trap (−70 °C), and stored at 20 °C in vacuum airtight packaging in a dark environment. Freeze-dried extracts were resuspended in physiological saline solution at a concentration of 1 mg/mL and filtered (pore filter 0.22 μm) for analysis and platelet aggregation assay.

### 2.4. Platelet Aggregation Assay

The inhibition of platelet aggregation was evaluated in a lumi-aggregometer (Chrono-Log) [5]. Thus, 480 μL of plasma rich in human platelets (2 × 10^5^ platelets/μL) was added to the reaction cuvette that was pre-incubated with 20 μL of extract (all extracts at a concentration of 1 mg/mL) or control for maximum platelet aggregation (0.9% saline). After 5 min of incubation, 20 μL of agonist (ADP, 4 μM) was added to initiate platelet aggregation, which was measured for 6 min. The results were expressed as a percentage of platelet aggregation. Platelets were obtained from 20 different donors and were used during a period of 2 h after blood extraction. All volunteers signed informed consent. The maximum platelet aggregation of the controls (without extracts) was 87 ± 6%. This study was conducted following the Declaration of Helsinki and informed consent was obtained for experimentation with human subjects.

### 2.5. Identification and Quantification of Bioactive Compounds by HPLC-MS

#### 2.5.1. Phenolic Compounds

Phenolic compounds were analyzed following the method of Torres et al., [36] with modifications. Briefly, 350 mg samples of each freeze-dried extract were weighed, ground, and mixed with 5 mL of 75% methanol and 100 µL of internal standard (naringenin, Sigma-Aldrich). Samples were centrifuged at 11,180× *g* at 10 °C for 10 min (International Equipment Company IEC, Centra MP4R, Boston, MA, USA). The supernatant was removed and diluted with 20 mL HPLC-grade water (Merck, Darmstadt, Germany). Simultaneously, extraction column C18 (No. 12102052, Agilent Technologies, Palo Alto, CA, USA) was activated with 1 mL of 75% methanol. Briefly, 5 mL HPLC-grade water was added to the C18 column and allowed to drain until dry to remove solvent residues. Samples were eluted using a vacuum pump (Welch 2545C/02, Mount Prospect, IL, USA) and a vacuum trap (20–40 kPa). Then, samples inside C18 were allowed to elute until completely drained using 2 mL of pure propanol to remove concentrated sample-specific phenolic compounds and collected in 4 mL test tubes. N_2_ gas was used to dry samples that were reconstituted in 200 µL of pure methanol, mixed, and sonicated (Bandelin Sonorex TK52H, Berlin, Germany) for 1 min. Samples were analyzed using the ultra-high performance liquid chromatography (UHPLC) Dionex UltiMate 3000 chromatography system (Thermo Scientific, Waltham, MA, USA) equipped with a refrigerated autosampler. The samples were injected into the Hypersil Gold C18 column (Thermo Fisher Scientific, Bremen, Germany) using 1 µL of samples and a gradient sample with 75% (*v*/*v*) acetonitrile, 24.5% (*v*/*v*) water, and 0.5% (*v*/*v*) formic acid (solution A) and 5% (*v*/*v*) acetonitrile, 94.5% (*v*/*v*) water, and 0.5% (*v*/*v*) formic acid (solution B) with a flow rate of 300 µL/min. Gradient elution conditions were set as follows: initial 0–1 min (10% B), 1–5 min (10% B), 5–10 min (30% B), 10–18 min (100% B), and 18–24 min (0% B), with final cleaning and reconditioning of the column. Mass quantification was carried out with an Exactive Plus Orbitrap spectrometer (Thermo Scientific) equipped with an electrospray interface operating with negative ionization mode, and data were processed using the Xcalibur 2.1 software (Thermo Scientific). Mass spectrometry conditions were 2500 spray volts, vaporization temperature of 350 °C, sheath gas pressure of 40 arbitrary units (a.u.), auxiliary gas pressure of 10 a.u., and capillary temperature of 35 °C. N_2_ was used as the collision gas and all values were normalized to 350 mg/dry weight. Phenolic compounds were identified and quantified using suitable standards (Extrasynthese, Lyon, France), which were prepared as 1 mg/mL stock solutions in methanol and stored at −80 °C for up to 1 month in dark conditions. All results were expressed in mg/100 g dry weight.

#### 2.5.2. Carotenoids Compounds

Fuentes et al. (2013) procedures were followed with modifications [37]. Briefly, 0.71 mL of 2:1 solution (pure acetone and 0.2 M HEPES buffer (pH 7.7)) was mixed with 500 mg of the freeze-dried extract sample and then agitated and centrifuged at 11,180× *g* for 5 min. The supernatant was separated; and the pellet was mixed with 0.71 mL of 2:1 solution (pure acetone and 0.2 M HEPES buffer (pH 7.7)), then stirred, and centrifuged to obtain the supernatant, which was then deposited together with the previous one. After that, 1 mL of pure acetone was added again to the pellet and centrifuged, the supernatant was deposited in the same tube, 1 mL of pure acetone was added to the pellet and it was stirred and centrifuged, the supernatant was deposited in the same tube, and 0.75 mL of pure hexane was added to the pellet, which was stirred and centrifuged. The supernatant was transferred to the same tube and 0.75 mL of hexane was added and centrifuged. The sample was recovered and evaporated with N_2_ gas; and the obtained sample was reconstituted, microfiltered, and stored in vials. UHPLC-MS was used for quantification with lycopene and β-carotene standards. Results were expressed in mg/100 g dry weight.

#### 2.5.3. Nucleosides Compounds

The method used was performed considering Dudley and Bond recommendations [38]. Briefly, 1 g of the freeze-dried extract was mixed with 20 mL of ultra-pure water and then homogenized in a mortar. The extract was sonicated with ultrasound (VWR 97043-962, Leicestershire, UK) at 35 kHz for 30 min, then centrifuged at 11,180× *g* for 10 min at 10 °C, and filtered at 0.22 µL with a microfilter disc (Millex-GN PTFE, Merck Millipore, Darmstadt, Germany). Quantification was performed by UHPLC-MS. An ESI injector with a positive charge at 35 kV, a capillary temperature of 350 °C, a flow of 250 µL were used. Mobile phase A was a solution of 10 mM ammonium acetate with 0.8% acetic acid and mobile phase B was a solution of acetonitrile with 0.1% acetic acid. A gradient of 10% at the first 6 min, then 6 min at 50% A, and then return in 6 min at 10% A was used. For quantification, adenosine, guanosine, and inosine standards were used in a curve of 0.05–1.5 µg/µL. Results were expressed in µg/100 g dry weight.

## 3. Competitive Study

The platelet anti-aggregant activity was compared with commercial cardioprotective products sold in the Chilean market (M1: CardioSmile, Nutrartis S.A., Providencia, Chile; M2: UltraPure Omega 3, Unicaps, Brea. CA, USA; M3: Eykosacol, Procaps S.A., Barranquilla, Colombia; M4: Benexia, Functional Products Trading S.A., Vitacura, Chile; and M5: Maqui Berry, Nativ for Life, Santiago, Chile). Liquid products (M2, M3, and M4) were evaluated directly on platelet aggregation at 1 mg/mL, while the solid product (M5) was dissolved in physiological serum in a final study concentration of 1 mg/mL. For the M1 product (given its milky consistency that affects the turbidity of the plasma in the platelet aggregation test), an aqueous extract was obtained. Thus, the product was mixed with water in a 1/8 ratio and centrifuged at 11,180× *g* for 5 min to obtain the supernatant that was freeze-dried and kept at −20 °C until use. Before the platelet aggregation assay, the aqueous extract of product M1 was resuspended in physiological saline solution at a concentration of 1 mg/mL.

## 4. Statistical Analysis

Data were expressed as mean ± standard deviations and analyzed by the Prism 6.0 software (GraphPad Inc., La Jolla, CA, USA). All measurements were made from six different donors. Before performing the statistical analysis, it was necessary to know if the results met with a normal distribution or not. Thus, using a significance level of 5% and according to Kolmogorov statistic with a *p*-value of 0.003, the results of platelet aggregation showed a non-normal distribution. The results of percentage of platelet aggregation were analyzed using non-parametric Kruskal–Wallis test, and subsequently analyzed by Dunn’s test, used as a post-test, to establish significant differences between each extract with respect to control (*p*-value < 0.05).

## 5. Results and Discussion

### 5.1. Extraction Yield

Table 1 shows the extraction yield of different types of tomato pomace extracts. From 5 g of each type of tomato pomace sample, 0.023–0.22 g of a dry extract was obtained. Freeze drying allowed obtaining extracts (e.g., AWTPE3) that are considered microbiologically safe [5,23]. The process of ultrasound extraction produces a phenomenon called cavitation which generates physical, chemical, and mechanical effects responsible for the cellular wall disruption of the vegetal matrix [39,40]. According to many authors (Al-Dhabi et al., 2017; Chemat et al., 2017; Contamine et al., 1995; Delgado-Povedano and de Castro, 2017; Mason et al., 1996; Rastogi, 2011), the association of different ultrasound extraction conditions (e.g., power, temperature, time, and solvent) may change the polarity and viscosity of the system, as well as the interaction between the solute and solvent. Thus, in this study, aqueous extracts (2.8 ± 1.1% m/m) showed higher extraction yields in comparison with ethanolic extractions (1.0 ± 0.5% m/m), and this was due to the polarity and viscosity of these solvents. According to Chemat et al. (2017) and other studies, variations in viscosity, although small, may induce resistance to ultrasound waves and it may affect the extraction efficiency of the solvent system [39,41,42].

The cavitation phenomena also cause a temperature rise with the extraction time, increasing the extraction yield of phenolic and nucleosides compounds by their higher solubility and diffusion. However, when a higher extraction time exposes these compounds at high temperatures for a long time, it may promote its degradation by oxidation mechanisms, consequently decreasing the extraction yield [41,42,43]. Therefore, in this study, cycle 3 showed promising potential to obtain high extraction yields, since AWTPE3 preliminarily presented 3.45% (m/m). This could be explained by the higher value of soluble solids observed for AWTPE3 (6.00 °Brix) compared with whole ground dried tomato pomace (1.67 °Brix, performed in 1 g of sample powder dissolved in 10 mL ultrapure water). The latter can be explained due to mechanical effects of ultrasound that triggered the release of water-soluble compounds such as polysaccharides, polyphenols, and nucleosides from their matrices by disrupting them from cellular tissues [5,6,23,33,34,44,45]. Ultrasound is also an effective method to improve fractioning of water-soluble compounds from tomato pomace fiber, since the latter was separated as a precipitate after centrifugation [5,23]. 

### 5.2. Platelet Anti-Aggregant Activity 

We observed that the non-ultrasound-assisted aqueous extraction of whole tomato pomace affected the platelet anti-aggregant activity of the extract (64 ± 11% vs. control 87 ± 6%, not significant). The time of sonication and solvent used in the extraction had a significant role in platelet anti-aggregant activity [46]. As is shown in the Table 1, the extracts with significant antiplatelet activity, with respect to control (87 ± 6%), were AWTPE3 (32 ± 9%, *p* < 0.01), EWTPEC3 (32 ± 9%, *p* < 0.01), ASTPEC6 (26 ± 6%, *p* < 0.001), ESTPEC6 (29 ± 7%, *p* < 0.01), ASEC1 (29 ± 8%, *p* < 0.01), ASEC3 (34 ± 2%, *p* < 0.05), AE5TPSC1 (27 ± 12%, *p* < 0.001), and EE5TPSC1 (29 ± 12%, *p* < 0.01). These levels of inhibition of platelet aggregation induced by ADP were different, depending on ultrasound cycle times, tomato pomace composition, and solvent used. Thus, the sonication cycles only improved the platelet anti-aggregant activity in the seedless extracts. Six cycles of sonication of 20 min each significantly improved the platelet anti-aggregant activity of aqueous (ASTPEC6 26 ± 6%, *p* < 0.001) and ethanolic (ESTPEC6, 29 ± 7%, *p* < 0.01) seedless tomato pomace extracts. Tomato seeds are composed with proteins (35.5%), fiber (13.2%), fatty acids (30%), ash (3%), and also essential amino acids (except tryptophan), so they could be used in several applications, for example, as an enrichment for cereal products and foods low in lysine [22,47].

For aqueous seed extracts and aqueous 50% tomato pomace/50% seed extracts, the best activity was achieved using one ultrasound cycle (ASEC1, 29 ± 8%, *p* < 0.01 and AE5TPSC1, 27 ± 12%, *p* < 0.001), while antiplatelet activity decreased with an increase to six sonication cycles (ASEC6, 40 ± 7% and AE5TPSC6, 54 ± 9%, not significant). On the other hand, three-cycle sonication allowed the best significant activity of the extracts obtained from aqueous whole tomato pomace (AWTPE3, 32 ± 9%, *p* < 0.01) and ethanolic whole tomato pomace (EWTPEC3, 32 ± 9%, *p* < 0.05). Although, in a previous study, the ultrasound extraction time used by the authors was 5 min [10], in this study ultrasound cycle was increased to 20 min to achieve a better extraction of compounds responsible for platelet anti-aggregant activity. Furthermore, 5 min of ultrasound extraction achieved an aggregation of 55% in aqueous tomato pomace extract [10], while the 20-min cycle improved the activity of the aqueous tomato pomace extract by lowering maximum aggregation to 46 ± 12% (AWTPE1).

Based on the results of yield and platelet anti-aggregant activity, it seemed that the most suitable extract was AWTPE3 (yield, 3.45% and platelet aggregation, 32 ± 9%, *p* < 0.01). Meanwhile, ASTPEC6 (platelet aggregation, 26 ± 6%, *p* < 0.001) had a poor yield of 0.80% and showed no statistically significant differences in platelet anti-aggregant activity when compared to AWTPE3. 

### 5.3. Identification of Bioactive Compounds 

Table 2 shows the amounts of bioactive compounds by HPLC-MS on aqueous extracts (whole tomato pomace, seed, and seedless) obtained from the same original matrix. Since tomato pomace is mainly composed of peels and seeds, we compared the bioactive compounds of AWTPE3 (aqueous whole tomato pomace), previously reported [5], with two other aqueous extracts obtained with the same sonication cycles—ASTPEC3 (aqueous seedless tomato pomace) and ASEC3 (aqueous seeds).

Several studies have observed that ultrasound is effective in assisting the extraction of bioactive compounds such as nucleosides, polyphenols, and carotenoids from different plant byproducts, including tomato pomace [5,27,48,49]. In comparison with unprocessed tomatoes and on a dry weight basis, tomato pomace contained significantly lower amounts of lycopene and increased amounts of β-carotene, tocopherols, sterols, terpenes, and flavonoids (e.g., naringenin) [50]. Thus, in this study higher concentrations of flavonoids (procyanidin B2, kaempferol-3-*O*-glucoside, and kaempferol) were observed for AWTPE3 in comparison with whole dried ground tomato pomace [5]. These results demonstrated that the ultrasound-assisted extraction and concentration using freeze drying resulted in a greater concentration of flavonoids in AWTPE3 [5]. A similar result was obtained by Navarro-González et al., where the best extraction of phenolic compounds (e.g., rutin, naringenin, and chlorogenic acid derivatives) was with ultrasonic assistance [51]. This concentration of compounds (e.g., kaempferol-3-*O*-glucoside) in AWTPE3 may be due to the fact that tomato pomace is composed of pulp residues, in addition to peels and seeds [52].

ASEC3 and ASTPEC3 had different levels of polyphenols because they were lost simply by removing their peels or seeds. Thus, the removal of the peels represented a loss of lycopene (80%), β-carotene (57%), and phenolic compounds (63%). Meanwhile, the elimination of seeds had a greater impact on polyphenols with a loss of 63% [53]. ASEC3 showed higher concentrations of flavonoid compounds than ASTPEC3, except for quercetin, which was detected in tomato peels in higher concentrations than seeds, according to literature [44]. In this context, it has been described that seed aqueous lyophilized extract from tomato contains 20,657 mg/100 g phenolic compounds, with the highest amounts of quercetin-3-*O*-sophoroside, kaempferol-3-*O*-sophoroside, and isorhamnetin-3-*O*-sophoroside [54]. However, ASEC3 did not present concentrations of kaempferol and quercetin, suggesting differences due to an extraction procedure effect. ASTPEC3 did not show concentrations of coumaric acid, apigenin-7-*O*-glucoside, kaempferol-3-*O*-glucoside, genistein, kaempferol, daidzein, quercitrin, and epicatechin, suggesting that these compounds were not effectively recovered from tomato peels by ultrasound-assisted extraction (Table 2). Chlorogenic acid, which is usually predominant in tomato byproducts, was not detected or quantified by chromatography in any of the samples. The latter could be explained due to low stability of this molecule during storage of dehydrated tomato byproducts [55]. 

The data available in the literature suggest a potential therapeutic effect of several flavonoids against CVD through the inhibition of platelet aggregation, such as coumaric acid [56], phloretin [57], procyanidin B2 [58], kaempferol-3-*O*- glucoside [59], and epicatechin [60]. The presence of these compounds in greater amounts in AWTPE3 and ASEC3 than in ASTPEC3 correlated with a better capacity to inhibit platelet aggregation. Several mechanisms of antiplatelet action of polyphenols have been reported, including inhibition of the arachidonic acid pathway, suppression of cytoplasmic increase in Ca^2+^, degradation blockage, αIIbβ3 integrin-mediated signaling, inhibition of secretion of platelet granules, and inhibition of thromboxane formation [61,62]. The ultrasound was able to increase the extraction of adenosine in AWTPE3 when compared with whole dried ground tomato pomace from 6.32 µg/100 g to 42.90 µg/100 g, almost 6 times more [5]. However, the extraction of inosine and guanosine was not affected by ultrasound. The results obtained from the quantification of nucleosides (adenosine, inosine, and guanosine) demonstrated that AWTPE3 presented greater amounts of these compounds compared with ASEC3 and ASTPEC3 [5]. Authors involved in this present work have previously observed that adenosine is concentrated in higher amounts in the aqueous extract from ripe tomato pulp than whole tomato pomace and peels extracts [9]. The presence of inosine in significant concentrations in ASEC3, but not in ASTPEC3, suggests that this compound is available only in tomato seeds [6,63]. In part, this could explain why ASEC3 had better platelet anti-aggregant activity than ASTPEC3, since inosine has been reported to inhibit platelet aggregation, significantly preventing in vivo thrombus formation [34]. Both extracts (ASEC3 and ASTPEC3) presented close concentrations of guanosine and adenosine, the latter had no significant amount detected. The present research group isolated and identified adenosine and guanosine as bioactive compounds in tomato with a potent platelet anti-aggregant activity [9,33,34].

The results showed that ultrasound was not efficient to extract carotenoids in the conditions established. Lycopene was solely quantified in ASTPEC3. Lycopene is found mainly in the peels and acts as a red pigment and, although resistant to thermal processing, it is very susceptible to light degradation [64]. To determine this compound, a prior extraction process must be carried out, which requires conditions of absence of light to avoid degradation, which makes its quantification very complicated and delicate. In the case of β-carotene, a higher concentration was found in whole dried ground tomato pomace powder in comparison with the other samples. According to literature, most non-polar solvents have higher extraction yield of carotenoids than aqueous extracts, since they are lipophilic compounds that are soluble in organic solvents, such as acetone, alcohol, ethyl ether, chloroform, and ethyl acetate. Therefore, large concentrations of carotenoids in the aqueous extract of tomato pomace were not expected.

### 5.4. Competitive Study

**Figure 1:**
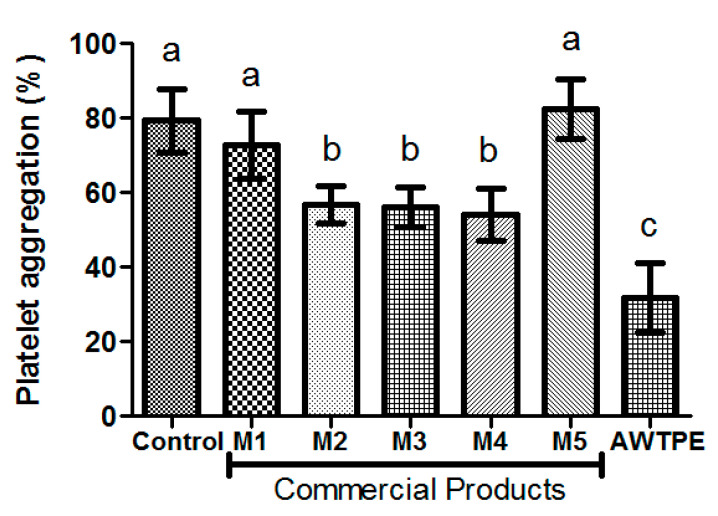
Platelet anti-aggregant activity of commercial products. Different letters indicate a significant difference with a *p* value < 0.05. AWTPE: aqueous whole tomato pomace extract cycle 3; M1: CardioSmile, Nutrartis S.A., Chile; M2: UltraPure Omega 3, Unicaps CA, USA; M3: Eykosacol, Laboratorio Procaps S.A., Colombia; M4: Benexia, Functional Products Trading S.A., Chile; and M5: Maqui Berry, Nativ for Life, Chile.

In the current market, there are cardioprotective products like phytosterols, polyphenols, and omega-3 [65]. Platelet anti-aggregant activity of commercial products was compared with that of AWTPE3 (Figure 1). In the presence of M2 (platelet aggregation, 57 ± 5%), M3 (platelet aggregation, 56 ± 5%), and M4 (platelet aggregation, 54 ± 7%), platelets showed a significant decrease in aggregation compared with the control of maximum platelet aggregation (79 ± 8%). Products M1 and M5 did not show inhibition of platelet aggregation. AWTPE3 presented a significant difference compared with the control and the commercial products M2, M3, and M4.

## 6. Conclusions

**Figure 2:**
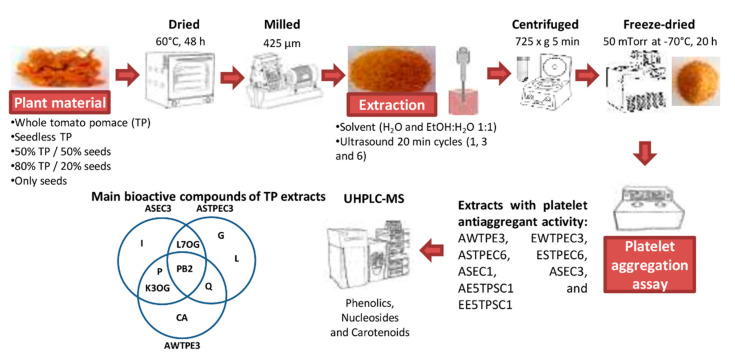
Platelet anti-aggregant activity and bioactive compounds of ultrasound-assisted extracts from whole and seedless tomato pomace. AE5TPSC1: aqueous extract 50% tomato pomace/50% seed cycle 1, ASEC1: aqueous seed extract cycle 1, ASEC3: aqueous seed extract cycle 3, ASTPEC3: aqueous seedless tomato pomace extract cycle 3, ASTPEC6: aqueous seedless tomato pomace extract cycle 6, AWTPE3: aqueous whole tomato pomace extract cycle 3, EE5TPSC1: ethanolic extract 50% tomato pomace/50% seed cycle 1, ESTPEC6: ethanolic seedless tomato pomace extract cycle 6, EWTPEC3: ethanolic whole tomato pomace extract cycle 3, CA: coumaric acid, G: guanosine, I: inosine, K-3-O-G: kaempferol-3-O- glucoside, L: lycopene, L7OG: luteolin-7-O- glucoside, P: phloretin, PB2: procyanidin B2, Q: quercetin.

Currently, tomato pomace is considered as waste by the food industry; however, in this study, we showed that ultrasound-assisted extracts from this byproduct had significant platelet anti-aggregant activity. The levels of inhibition of platelet aggregation were different, depending on ultrasound cycle times, tomato pomace composition (whole, seedless, and seed), the solvent used, and the flavonoid and nucleoside content (Figure 2). In this context, the ultrasound-assisted extraction improved extraction yields, showing significant recovery of bioactive compounds from tomato pomace and increased platelet aggregation inhibition.

## Figures and Tables

**Table 1:** Extraction yield and platelet anti-aggregant activity of different types of tomato pomace extracts.

Type of Extracts	Code	Yield (%)	Platelet Aggregation (%)
Aqueous whole tomato pomace extract cycle 1	AWTPE1	3.57	46 ± 12
Aqueous whole tomato pomace extract cycle 3	AWTPE3	3.45	32 ± 9 **
Aqueous whole tomato pomace extract cycle 6	AWTPE6	2.13	48 ± 3
Ethanolic whole tomato pomace extract cycle 1	EWTPEC1	0.82	52 ± 8
Ethanolic whole tomato pomace extract cycle 3	EWTPEC3	0.68	32 ± 9 **
Ethanolic whole tomato pomace extract cycle 6	EWTPEC6	0.57	51 ± 9
Aqueous seedless tomato pomace extract cycle 1	ASTPEC1	1.31	61 ± 7
Aqueous seedless tomato pomace extract cycle 3	ASTPEC3	1.87	46 ± 9
Aqueous seedless tomato pomace extract cycle 6	ASTPEC6	0.80	26 ± 6 ***
Ethanolic seedless tomato pomace extract cycle 1	ESTPEC1	ND	ND
Ethanolic seedless tomato pomace extract cycle 3	ESTPEC3	0.59	45 ± 9
Ethanolic seedless tomato pomace extract cycle 6	ESTPEC6	0.46	29 ± 7 **
Aqueous seed extract cycle 1	ASEC1	2.51	29 ± 8 **
Aqueous seed extract cycle 3	ASEC3	1.60	34 ± 2 *
Aqueous seed extract cycle 6	ASEC6	3.45	40 ± 7
Ethanolic seed extract cycle 1	ESEC1	0.48	38 ± 4
Ethanolic seed extract cycle 3	ESEC3	1.44	47 ± 8
Ethanolic seed extract cycle 6	ESEC6	1.27	52 ± 12
Aqueous extract, 50% tomato pomace/50% seed cycle 1	AE5TPSC1	2.96	27 ± 12 ***
Aqueous extract, 50% tomato pomace/50% seed cycle 3	AE5TPSC3	4.43	36 ± 8
Aqueous extract, 50% tomato pomace/50% seed cycle 6	AE5TPSC6	2.61	54 ± 9
Ethanolic extract, 50% tomato pomace/50% seed cycle 1	EE5TPSC1	1.11	29 ± 12 **
Ethanolic extract, 50% tomato pomace/50% seed cycle 3	EE5TPSC3	ND	ND
Ethanolic extract, 50% tomato pomace/50% seed cycle 6	EE5TPSC6	ND	ND
Aqueous extract, 80% tomato pomace/20% seed cycle 1	AE8TPSC1	2.94	41 ± 7
Aqueous extract, 80% tomato pomace/20% seed cycle 3	AE8TPSC3	4.25	40 ± 11
Aqueous extract, 80% tomato pomace/20% seed cycle 6	AE8TPSC6	4.08	38 ± 11
Ethanolic extract, 80% tomato pomace/20% seed cycle 1	EE8TPSC1	2.29	39 ± 15
Ethanolic extract, 80% tomato pomace/20% seed cycle 3	EE8TPSC3	1.07	52 ± 16
Ethanolic extract, 80% tomato pomace/20% seed cycle 6	EE8TPSC6	1.28	39 ± 12
Non-ultrasound-assisted aqueous whole tomato pomace extract			64 ± 11
Control (maximum platelet aggregation)			87 ± 6

Platelet aggregation data were expressed as mean ± standard deviations (SD). Statistically significant difference in platelet aggregation (%) is considered with * *p* < 0.05, ** *p* < 0.01, and *** *p* < 0.001, analyzed with Dunn’s test with respect to control. ND: not determined. Not determined (ND) extracts correspond to extracts that were generated, but obtained at a very low yield, preventing them to be weighed and evaluated. Cycle 1: 20 min, cycle 3: three cycles of 20 min each; and cycle 6: six cycles of 20 min each.

**Table 2:** Identification and quantification of bioactive compounds by HPLC-MS.

	Samples		
Compounds **	AWTPE3 *	ASEC3	ASTPEC3
*Flavonoids (mg/100 g Dry Weight)*			
Gallic acid	0.83	6.94	0.53
Ferulic acid	2.44	9.08	3.68
Coumaric acid	88.56	2.58	<0.001
Phloridzin	4.71	1.35	2.62
Phloretin	97.31	26.72	1.71
Procyanidin B2	1868.49	76.62	27.95
Apigenin-7-*O*-glucoside	<0.001	0.196	<0.001
Kaempferol-3-*O*-glucoside	2032.58	415.39	<0.001
Luteolin-7-*O*-glucoside	63.34	55.77	57.63
Genistein	<0.001	0.196	<0.001
Kaempferol	77.09	<0.001	<0.001
Daidzein	<0.001	0.02	<0.001
Quercetin	408.23	<0.001	7.74
Quercitrin	1.96	0.003	<0.001
Rutin	0.262	0.065	0.012
Epicatechin	0.131	0.026	<0.001
*Nucleosides (µg/100 g Dry Weight)*			
Adenosine	42.90	<0.001	<0.001
Inosine	57.20	42.21	<0.001
Guanosine	20.97	7.44	7.26
*Carotenoids (mg/100 g Dry Weight)*			
Lycopene	<0.001	<0.001	7.74
β-Carotene	55.5	4.3	3.4

* Data reported in Palomo et al., (2019) [5]. *p* < 0.001 indicates concentrations close to the limit of detection. AWTPE3: aqueous whole tomato pomace extract cycle 3, ASEC3: aqueous seed extract cycle 3, and ASTPEC3: aqueous seedless tomato pomace extract cycle 3. ** Twenty-one flavonoid molecules were analyzed and sixteen molecules were detected by chromatography. Three nucleoside molecules were analyzed and detected by chromatography. Two carotenoid molecules were analyzed and detected by chromatography.
